# Factors affecting caregivers’ participation in support groups for people living with HIV in Tanzania

**DOI:** 10.3389/fpubh.2023.1215219

**Published:** 2023-09-15

**Authors:** Levina Kikoyo, Amon Exavery, John Charles, Akwila Temu, Asheri Barankena, Amal Ally, Remmy Mseya, Tumainiel Mbwambo, Rose Fovo, Aidan Tarimo, Godfrey Martin Mubyazi, Marianna Balampama, Erica Kuhlik, Tom Ventimiglia, Elizabeth Lema

**Affiliations:** ^1^Pact Tanzania, Dar es Salaam, Tanzania; ^2^National Institute for Medical Research (NIMR), Dar es Salaam, Tanzania; ^3^Pact USA, Washington, DC, United States; ^4^USAID, Dar es Salaam, Tanzania

**Keywords:** support groups for people living with HIV, membership, HIV, caregivers, Tanzania

## Abstract

**Introduction:**

Support groups for people living with HIV (PLHIV) are essential for increasing adherence, retention, addressing their psychosocial needs and improving patient literacy. However, factors that influence participation of caregivers living with HIV (LHIV) in these groups are scarcely documented, particularly for those caring for orphans and vulnerable children (OVC).

**Methods:**

This study used baseline data collected between 1st October 2021 and 30th September 2022 from the PEPFAR/USAID-funded Adolescents and Children HIV Incidence Reduction, Empowerment and Virus Elimination (ACHIEVE) project in Tanzania to investigate factors that affect participation of caregivers LHIV in support groups for PLHIV. A total of 74,249 HIV-positive OVC caregivers who were already receiving antiretroviral therapy (ART) and had a confirmed care and treatment centre identification number were included in the analysis. Factors affecting group participation were identified through multilevel analysis using multivariable mixed-effects logistic regression.

**Results:**

Results showed that 84.2% of the caregivers were participants in the support groups for PLHIV. Their mean age was 36 years, and the majority (82.1%) were female. Multivariable analysis revealed that participation in the groups was more likely among caregivers living in urban areas (aOR = 1.39 [1.24, 1.55]), with primary education (aOR = 1.17 [1.07, 1.28]), and without disabilities (aOR = 0.62 [0.47, 0.82]). However, participation was less likely among widowed (aOR = 0.91 [0.84, 0.999]), single or unmarried (aOR = 0.86 [0.78, 0.95]), and those with secondary education or higher levels than never attended (aOR = 0.69 [0.60, 0.80]), moderate hunger (aOR = 0.86 [0.79, 0.93]), and those aged 30 years or older (*p*< 0.001).

**Discussion:**

A sizeable proportion (15.8%) of the caregivers were not in support groups for PLHIV, ranging from 12.3% among those in households with severe hunger to 29.7% among disabled ones. The study highlights the need for tailored interventions to increase participation in support groups for PLHIV, particularly for caregivers who are disabled, live in rural areas, are older, widowed, and/or unmarried, and those in poor households.

## 1. Introduction

Substantial evidence shows that support groups for people living with the Human Immunodeficiency Virus (PLHIV) are crucial in increasing adherence to and retention in HIV care and treatment services, addressing literacy and psychosocial needs (1–5), and managing stress and stigma and improving wellbeing. They act as a space to practice and form new behaviors that are necessary for coping and living healthy with the virus (6). PLHIV support groups are voluntary and designed to fulfill a common purpose to address a shared challenge (e.g., HIV) that alters the normal course of life (1, 7). In sub-Saharan Africa, support groups are extensively accepted as an integral part of HIV care and treatment programs (3, 6) due to their significant contribution to increasing PLHIV’s wellbeing as a result of lower HIV-related illnesses and deaths among participants than non-participants (8).

In Tanzania, support groups for PLHIV exist, and consist of three age-appropriate peer groups: (a) children (age 0–14 years), (b) adolescents or teen clubs (age 15–17 years), and (c) adults (age ≥ 18 years). The support groups for PLHIV are created on the basis that PLHIV health outcomes will improve when clinical services received are complemented by quality social support and age-appropriate information and attention to treatment, adherence, HIV status disclosure, positive living, and life skills for healthy living and overall welfare (4). All PLHIV are informed about and encouraged to join the support groups for PLHIV (9) to easily gain access to treatment support and literacy as well as socialize and normalize their experience of living with HIV (4, 5).

Despite the known importance of these groups, limited evidence exists on the factors that affect participation in the PLHIV support groups for different population groups such as HIV-positive caregivers of orphans and vulnerable children (OVC) (1–3, 5). Caregivers are considered a unique population with special needs because they care for their loved ones and other individuals who cannot live independently, but they are not prepared or equipped for the caregiving job (10). Research evidence reveals that caregivers face significant economic, physical, behavioral, and psychological vulnerabilities in the caregiving process (10–12). These vulnerabilities are further compounded by HIV (13, 14). This ultimately increases their risk of mental health issues as well as chronic stress because the caregiving role alters their work, relationships, and friendships (15, 16). Indeed, a recent empirical study in India found that caregivers were more likely to have depression and poor self-rated health than non-caregivers (17).

It is evident that to achieve HIV epidemic control, it is critical to target everyone with key interventions such as the PLHIV support groups for better health outcomes. Given the voluntary nature of participation in these groups, it is crucial to target PLHIV at a higher risk of treatment interruption (7). Therefore, this study aimed to identify factors associated with participation in support groups for PLHIV among HIV-positive caregivers of OVC in Tanzania, with the goal of providing targeted support to those who may benefit the most.

## 2. Materials and methods

### 2.1. Data source

This study utilized data from the Adolescents and Children, HIV Incidence Reduction, Empowerment, and Virus Elimination (ACHIEVE) project in Tanzania. ACHIEVE Tanzania is a four-year project (2020–2024), funded by the U.S. President’s Emergency Plan for AIDS Relief (PEPFAR) through the United States Agency for International Development (USAID). The project aims to strengthen the capacity of the national and community-level social services workforce, systems, and structures to provide quality services for OVC, at-risk adolescent girls and young women (AGYW), and PLHIV. Together with systems strengthening, the project provides direct services to OVC and beneficiaries of the Determined, Resilient, Empowered, AIDS-free, Mentored, and Safe (DREAMS) initiative in designated geographical areas (18). ACHIEVE is a global project; it is implemented in other countries, namely, Burundi, the Dominican Republic, Namibia, Nigeria, Rwanda, South Africa, South Sudan, and Zambia by Pact and its consortium partners – Jhpiego, Palladium, No Means No Worldwide (NMNW), and WI-HER (19).

Data were collected by community case workers (CCWs) during screening and enrollment of beneficiaries into the ACHIEVE project from 1st October 2021 to 30th September 2022. CCWs are community-based volunteers who are trained in the provision of basic health and social welfare case management services. Beneficiaries were enrolled in the project if their household had at least one HIV-related vulnerability, including the presence of at least one child in the household who is living with HIV, the household having a child born or breastfed by a woman living with HIV, the presence of a child of a female sex worker (FSW) in the household, at least one child in the household having been or being abused, presence of a child of an HIV positive parent/caregiver in the household, and others.

### 2.2. Study area

**Figure 1:**
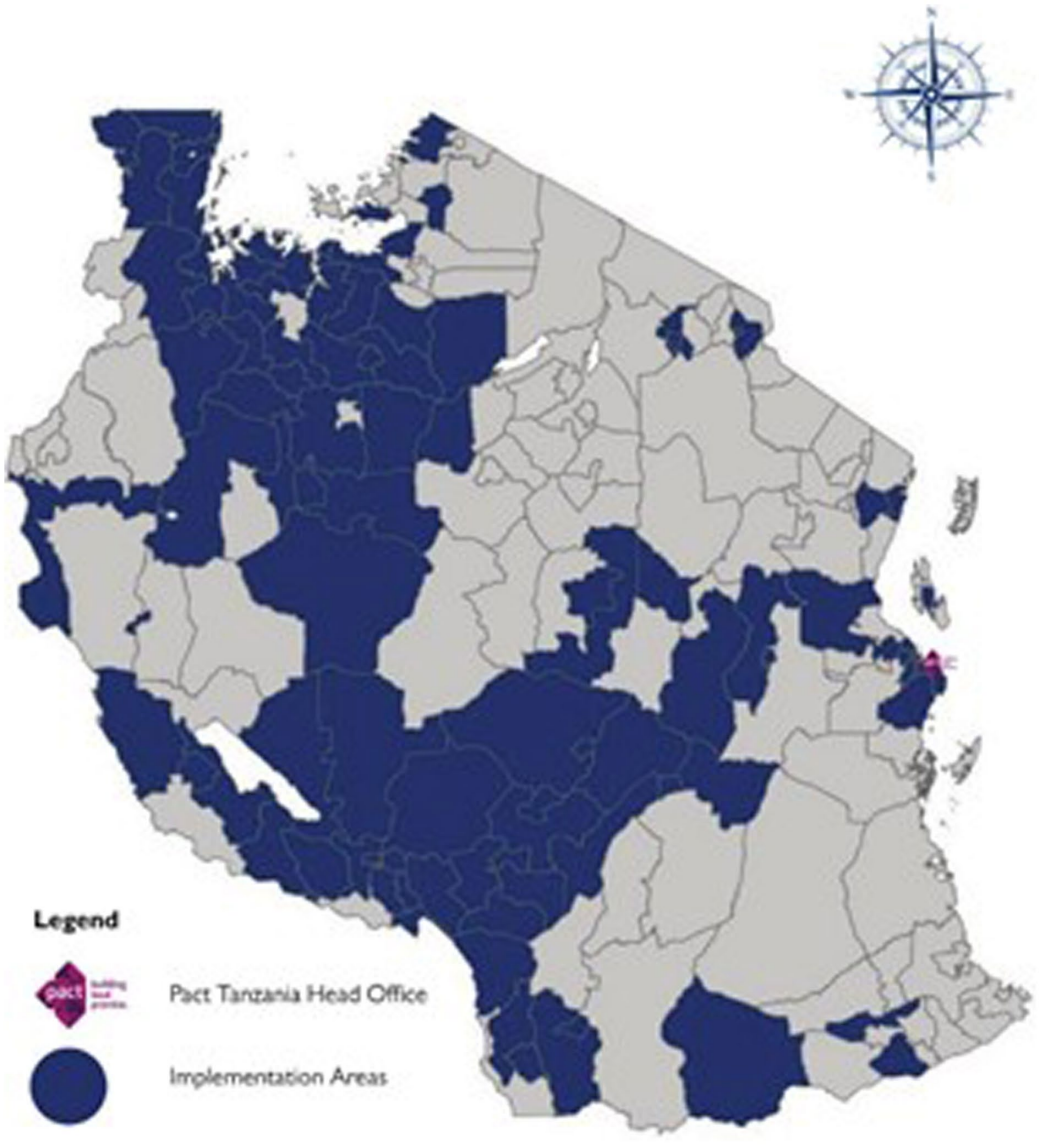
A map of Tanzania showing implementation areas of the ACHIEVE project in Tanzania between 1st October 2021 and 30th September 2022.

The data analyzed in this study are derived from beneficiaries in 95 district councils from 25 regions of Tanzania where enrollment activities for the ACHIEVE project had occurred from 1st October 2021 to 30th September 2022 (Figure 1). The regions are Arusha, Dodoma, Dar es Salaam, Geita, Iringa, Kagera, Katavi, Kigoma, Kilimanjaro, Mara, Mbeya, Mjini Magharibi in Zanzibar, Morogoro, Mtwara, Mwanza, Njombe, Pwani, Rukwa, Ruvuma, Shinyanga, Singida, Simiyu, Songwe, Tabora, and Tanga.

### 2.3. Study design

This study involves a secondary analysis of existing cross-sectional data collected as a part of screening and enrollment (i.e., baseline) of beneficiaries into the ACHIEVE project in Tanzania.

### 2.4. Study population

The study focuses on OVC caregivers age ≥ 18 years who were living with HIV and already receiving antiretroviral therapy (ART), with a confirmed Care and Treatment Centre (CTC)-issued identification number. In the ACHIEVE project, each enrolled household has one registered caregiver, who is a parent/guardian with the greatest responsibility for daily care and rearing of at least one OVC in a household (20). As other PLHIV in Tanzania, the caregivers involved in this study have access to all available services for PLHIV, including free ART (21), multi-month prescription and dispensing, and several others according to the National Guidelines for the Management of HIV and AIDS.

### 2.5. Variables

#### 2.5.1. Dependent variable

The primary dependent (outcome) variable for this study is the caregiver’s participation status in support groups for PLHIV at the time of enrolment in the ACHIEVE project. This variable is binary and coded “1” if the caregiver was actively attending or participating in the support groups for PLHIV, and “0” otherwise. The caregiver’s participation status is determined through their self-reports and verified through probes to confirm their membership in any PLHIV support group.

#### 2.5.2. Independent variables

Sociodemographic characteristics of the caregivers were included as independent variables. These were sex, age (in years), education, marital status, household hunger level, and wealth quintile. Other independent variables were the place of residence, whether the caregiver has health insurance, and caregiver physical or mental disability.

Household hunger level was measured using the Household Hunger Scale (HHS). In the process of determining the level of household hunger, the HHS operationalizes three questions: (1) In the past 4 weeks, how often was there ever no food to eat of any kind in your household because of lack of resources to get food?, (2) In the past 4 weeks, how often did any household member go to sleep at night hungry because there was not enough food?, and (3) In the past 4 weeks, how often did any household member go a whole day and night without eating anything? (22). These questions were included in the project’s family and child asset assessment (FCAA) tool, which was used to collect the data analyzed for this study. According to the HHS, households are classified into three major categories according to their level of hunger: (1) little to no hunger households, (2) moderate hunger households, and (3) severe hunger households. The HHS is a validated scale for cross-cultural use, especially in food-insecure settings (22).

Wealth quintile was determined using Principal Component Analysis (PCA) (23) of household-owned assets. The assets used in the PCA process were land, dwelling materials (i.e., brick, concrete, cement, aluminum, other), livestock (i.e., chicken, goats, cows, other), transportation assets (i.e., bicycle, motorcycle/moped, tractor, motor vehicle, other), and productive assets (i.e., sewing machine, television, couch/sofa, cooking gas, hair dryer, radio, refrigerator, blender, oven, other).

### 2.6. Statistical analysis

The data analysis for this study involved both descriptive and analytical techniques. The unit of data analysis was the individual caregiver. Descriptive statistics were used to generate frequencies through one-way tabulations. This process expanded into bivariate analysis, where the participation of caregivers in PLHIV support groups was cross tabulated against each independent variable using the Chi-Square ( $\chi^{2}$ ) test to determine associations. The factors associated with caregivers’ participation in the PLHIV support groups were identified using multivariable mixed-effects logistic regression model. This regression technique was appropriate because caregivers receiving services from the same CCW may be correlated with respect to participation in the groups. The data analysis was performed using Stata (version 17.0) statistical software, and statistical inference was based on a significance level of 5% (α = 0.05).

### 2.7. Ethical considerations

This study was conducted with ethics approval from the Medical Research Coordinating Committee (MRCC) of the National Institute for Medical Research (NIMR) in Tanzania: NIMR/HQ/R.8a/Vol.IX/4080. All respondents were recruited voluntarily into the ACHIEVE project after signing the statement of informed consent. The project data and associated documents were stored with maximum security and confidentiality. Access to the data was limited to a few staff and the datasets analyzed for the current study were anonymous.

## 3. Results

### 3.1. Characteristics of respondents

The present study was based on 74,249 caregivers living with HIV (LHIV) and receiving ART in Tanzania. Their mean age was 36 years, and the majority were women (82.1%). Most of the caregivers had primary education (83.5%), and the most common marital status was married or living with their partners (62.9%). Rural residences represented 57.8% of the caregivers, and only a small percentage had health insurance (12.2%). Less than 1% of the caregivers had mental or physical disabilities. In terms of food security, only 21.3% of the caregivers were in households with little to no hunger (food secure), while the rest were in moderate (74.7%) and severe (4.0%) hunger households. These characteristics are summarized in Table 1.

**Table 1:** Characteristics of respondents (*n* = 74,249).

	Does not participate in PLHIV support groups (*n* = 11,746)		Participates in PLHIV support groups (*n* = 62,503)		Total (*n* = 74,249)	
	*n*	%	*n*	%	*n*	%
Overall	11,746	100.0	62,503	100.0	74,249	100.0
**Sex**						
Female	9,694	82.5	51,294	82.1	60,988	82.1
Male	2,052	17.5	11,209	17.9	13,261	17.9
**Age**						
20–29 years	3,840	32.7	21,545	34.5	25,385	34.2
30–39 years	3,218	27.4	17,023	27.2	20,241	27.3
40–49 years	2,749	23.4	14,648	23.4	17,397	23.4
50–59 years	1,270	10.8	6,398	10.2	7,668	10.3
60+ years	669	5.7	2,889	4.6	3,558	4.8
Summary	$\bar{x\;}$ = 36.6; σ = 13.1		$\bar{x}$ = 35.9; σ = 12.8		$\bar{x}$ = 36.0; σ = 12.9	
**Education level**						
Never attended/pre-primary	1,483	12.6	7,333	11.7	8,816	11.9
Primary	9,587	81.6	52,404	83.8	61,991	83.5
Secondary+	676	5.8	2,766	4.4	3,442	4.6
**Marital status**						
Married or living together	7,185	61.2	39,545	63.3	46,730	62.9
Divorced or separated	1,948	16.6	11,256	18.0	13,204	17.8
Widow/widower	1,480	12.6	6,716	10.8	8,196	11.0
Single/unmarried	1,133	9.7	4,986	8.0	6,119	8.2
**Wealth quintile**						
Lowest	2,728	23.2	13,384	21.4	16,112	21.7
Second	2,668	22.7	12,299	19.7	14,967	20.2
Middle	2,438	20.8	14,627	23.4	17,065	23.0
Fourth	1,858	15.8	11,787	18.9	13,645	18.4
Richest	2,054	17.5	10,406	16.7	12,460	16.8
**Level of household hunger**						
Little to no hunger	2,351	20.0	13,495	21.6	15,846	21.3
Moderate hunger	9,029	76.9	46,398	74.2	55,427	74.7
Severe hunger	366	3.1	2,610	4.2	2,976	4.0
**Place of residence**						
Rural	7,646	65.1	35,257	56.4	42,903	57.8
Urban	4,100	34.9	27,246	43.6	31,346	42.2
**Disabled?**						
No	11,604	98.8	62,167	99.5	73,771	99.4
Yes	142	1.2	336	0.5	478	0.6
**Has health insurance?**						
No	10,223	87.0	54,991	88.0	65,214	87.8
Yes	1,523	13.0	7,512	12.0	9,035	12.2

$\bar{x}$ = mean or average, σ = standard deviation.

### 3.2. Participation in PLHIV support groups by background characteristics

Table 2 presents the results of the bivariate analysis examining the relationship between caregivers’ participation status (%) in PLHIV groups and their background characteristics. Overall, 84.2% of the caregivers were actively participating in support groups for PLHIV. However, the level of participation varied significantly across different background characteristics. For instance, there were significant differences in participation in the PLHIV groups based on age (*p* < 0.001), education level (*p* < 0.001), marital status (*p* < 0.001), wealth quintile (*p* < 0.001), level of household hunger (*p* < 0.001), place of residence (*p* < 0.001), disability status (*p* < 0.001), and whether the caregiver has health insurance (*p* = 0.004).

**Table 2:** Status of caregivers’ participation in support groups for PLHIV by background characteristics (*n* = 74,249).

	Number of respondents (*n*)	% Not in PLHIV support groups	% In PLHIV support groups	*p*-value
Overall	74,249	15.8	84.2	–
**Sex**				0.229
Female	60,988	15.9	84.1	
Male	13,261	15.5	84.5	
**Age**				<0.001
20–29 years	25,385	15.1	84.9	
30–39 years	20,241	15.9	84.1	
40–49 years	17,397	15.8	84.2	
50–59 years	7,668	16.6	83.4	
60+ years	3,558	18.8	81.2	
**Education level**				<0.001
Never attended/pre-primary	8,816	16.8	83.2	
Primary	61,991	15.5	84.5	
Secondary+	3,442	19.6	80.4	
**Marital status**				<0.001
Married or living together	46,730	15.4	84.6	
Divorced or separated	13,204	14.8	85.3	
Widow/widower	8,196	18.1	81.9	
Single/unmarried	6,119	18.5	81.5	
**Wealth quintile**				<0.001
Lowest	16,112	16.9	83.1	
Second	14,967	17.8	82.2	
Middle	17,065	14.3	85.7	
Fourth	13,645	13.6	86.4	
Richest	12,460	16.5	83.5	
**Level of household hunger**				<0.001
Little to no hunger	15,846	14.8	85.2	
Moderate hunger	55,427	16.3	83.7	
Severe hunger	2,976	12.3	87.7	
**Place of residence**				<0.001
Rural	42,903	17.8	82.2	
Urban	31,346	13.1	86.9	
**Disabled?**				<0.001
No	73,771	15.7	84.3	
Yes	478	29.7	70.3	
**Has health insurance?**				0.004
No	65,214	15.7	84.3	
Yes	9,035	16.9	83.1	

*p*-values are based on Pearson’s Chi-Square test.

### 3.3. Findings from multivariable analysis

**Figure 2:**
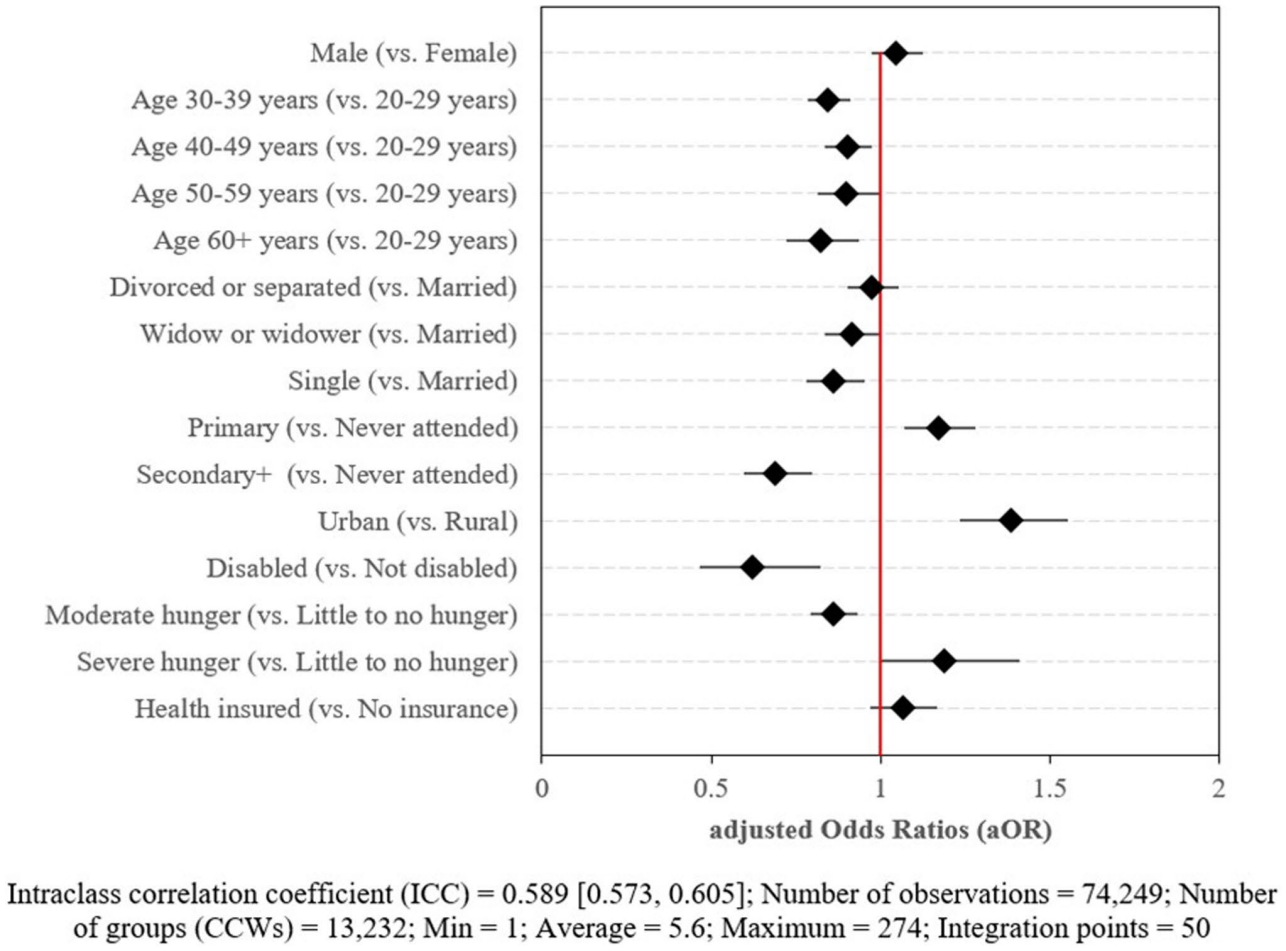
Multivariable mixed-effects logistic regression model of factors associated with caregivers’ participation in support groups for PLHIV in Tanzania (*n* = 74,249).

The multivariable analysis yielded adjusted odds ratios (aOR) and their corresponding 95% confidence intervals (CIs) for factors associated with caregivers’ participation in PLHIV, which are presented in Figure 2. All variables included in the model were adjusted for one another to account for confounding.

The results showed that male caregivers were as likely to participate in PLHIV groups as female caregivers (aOR = 1.05 [0.97, 1.13]). However, age was found to be negatively associated with participation in PLHIV groups. Specifically, caregivers in the age groups 30–39 years (aOR = 0.85 [0.79, 0.91]), 40–49 years (aOR = 0.90 [0.83, 0.97]), 50–59 years (aOR = 0.90 [0.81, 0.99]), and 60+ years (aOR = 0.82 [0.72, 0.94]) were less likely to participate in PLHIV groups than those in the age group 20–29 years.

Furthermore, caregivers with mental or physical disabilities were 38% less likely to participate in PLHIV groups compared to those without disabilities (aOR = 0.62 [0.47, 0.82]). Place of residence was also significantly associated with participation, with caregivers in urban areas being 39% more likely to participate in PLHIV groups than those in rural areas (aOR = 1.39 [1.24, 1.55]). Caregivers in households with moderate hunger were 14% less likely to participate in PLHIV groups compared to those in households with little to no hunger (aOR = 0.86 [0.79, 0.93]). However, caregivers in households with severe hunger were 19% more likely to participate in support groups compared to those with little to no hunger, although the association was only significant at the borderline (aOR = 1.19 [0.999, 1.41]).

With respect to marital status, widowed (aOR = 0.91 [0.84, 0.999]) and single or unmarried caregivers (aOR = 0.86 [0.78, 0.95]) were less likely to participate in the PLHIV groups compared to married caregivers. While caregivers with primary education were 17% more likely to participate in the PLHIV groups compared to caregivers who had never been to school (aOR = 1.17 [1.07, 1.28]), those with secondary education or more were 31% less likely to participate, compared to those that had never been to school (aOR = 0.69 [0.60, 0.80]).

Overall, the Intraclass Correlation Coefficient (ICC) was 58.9% (ICC = 0.589), which was the amount of variability in caregiver’s participation in the support groups for PLHIV due to being served by the same CCW.

## 4. Discussion

As already highlighted, this study aimed to investigate the factors associated with caregivers’ participation in support groups for PLHIV in Tanzania. It is crucial to understand the factors that influence participation in these groups, given the well-established benefits they provide (1–5). Identifying these factors can help develop targeted support strategies, particularly for those at higher risk of interruption in treatment. The study revealed that several factors were significantly associated with caregivers’ participation in the PLHIV support groups, including the level of household hunger, place of residence, physical or mental disability, age, marital status, and education level.

The study found that a majority (84.2%) of the caregivers were active participants in the PLHIV support groups, indicating generally a high level of engagement with the group support services. However, the study also revealed that a significant portion of the caregivers (15.8%) who were receiving ART lacked complementary support from the support groups for PLHIV. This proportion ranged from 12.3% among caregivers in households with severe hunger to 29.7% among those with physical or mental disabilities.

From the multivariable analysis, the higher odds of utilization of the support groups for PLHIV among caregivers living in urban areas compared to those in rural areas may be due to various factors including the fact that many PLHIV groups are established in selected high-volume facilities, which are more placed in urban than in rural areas. Also, it may be attributed to the shorter travel distances to the locations where PLHIV groups meet, and better transportation infrastructures which may be more so in urban than in rural areas. In contrast, rural caregivers may face transportation challenges, long distances to the health facilities or other locations where the groups meet, and inadequate resources for transportation (24, 25), which may limit their utilization of PLHIV support group services. These observations of higher likelihood of utilization of the support groups for PLHIV in urban than in rural areas have been found in similar studies (26–28). Strategies aimed at improving access to HIV support groups for rural caregivers may include provision of targeted support and resources for initiation of new support groups for PLHIV in health facilities (and communities) where they do not exist, and community-based initiatives to improve health literacy and awareness. It is crucial to address these barriers and ensure that rural residents have equal access to health services, including support groups for PLHIV.

Caregivers with physical or mental disabilities were significantly less likely to participate in the support groups for PLHIV than those without the disabilities. Although the disabled caregivers are few, accounting for less than 1% (*n* = 478) of the sample, it is already well recognized within the framework of universal health coverage (UHC) that service provision including those aimed at ending the AIDS epidemic, should leave no one behind (29). Therefore, specialized strategies should be devised to reach this group, given that disabled people are disadvantaged in many dimensions, including HIV status disclosure (30, 31), access to information, counseling and treatment (32, 33), obtaining HIV test results (32), ART utilization (33, 34), adherence to ART (35), and more (36–39). Further, stigma and discrimination associated with disability are widespread (40, 41), and can be significant barriers to service access in this group (30, 31, 42–44). Unfortunately, the adverse effects of disability are cascading, as one study found that children cared for by disabled caregivers are also less likely to access services (45) and caregiver’s poor health implies children’s poor health (46). Therefore, efforts to address inequalities in HIV care and treatment services should prioritize disability as one of the critical factors requiring urgent attention because such small groups are likely to be left behind in the future trajectories of the HIV epidemic.

Caregivers residing in households with moderate hunger were found to be significantly less likely to participate in the support groups for PLHIV than those in little to no hunger households (food secure). Other studies have also shown that caregivers have to face a lot of struggle with food insecurity due to the additional economic pressure of caregiving obligations (47–49). This suggests that such caregivers may prioritize securing food for their families, leaving them with limited time and resources to attend PLHIV support groups. Therefore, providing economic empowerment interventions, including social protection (e.g., direct food support) to address household hunger is likely to improve caregivers’ participation in PLHIV support groups, ultimately contributing to their overall wellbeing.

The present study observed that while caregivers with primary education were significantly more likely to participate in the PLHIV groups, those with secondary education or more were significantly less likely to participate compared to those with no formal schooling. While education can potentially reduce treatment misconceptions (50), and consequently improve service uptake (34), the likely mechanism underlying the observed direction of the association was unexpected. It is unclear whether those with higher levels of education may be engaged in commitments such as formal employments thereby lacking the time to participate in the groups, or if they feel superior to associate with less educated individuals. Further research is needed to explore and explain this association, so that barriers to participation can be addressed (e.g., by adjusting meeting times or places to cater to those with employment).

Marital status was found to be another significant factor affecting caregivers’ participation in support groups for PLHIV. Specifically, widowed, and unmarried or single caregivers were significantly less likely to participate in the groups compared to those who were married or living together with their spouses. This implies that marital unions may provide higher social support, which is associated with better HIV treatment adherence (51, 52), explaining the observed association. Therefore, it is crucial to provide targeted emotional or psychosocial support to widowed and unmarried or single caregivers to improve their participation in the PLHIV groups for their overall wellbeing.

Finally, the study found a negative association between age and participation in the PLHIV support groups in such a way that each of the age groups above 29 years had lower odds of participating in the groups compared to the age group 20–29 years. While this observation suggests the need to prioritize older caregivers in strategies to improve participation in the PLHIV groups, the underlying reason for this association remains unclear. Thus, further research is needed to investigate and clarify the factors contributing to this trend. Understanding these factors could help in developing targeted interventions to improve participation among older caregivers and ultimately improve their overall wellbeing.

### 4.1. Strengths and limitations

This study has several noteworthy strengths, including its large sample size which represents 25 out of 31 regions in Tanzania, thereby making it a nationally representative study. The use of standardized tools for collection of data helped minimize information bias and robust statistical techniques were utilized to identify factors associated with participation in the PLHIV groups, including multivariable analysis that accounted for the clustering of the caregivers at the CCW level.

However, there are some limitations to consider. Caregivers’ participation in PLHIV groups was self-reported, giving room to recall and or desirability bias which is typically associated with self-reported data (53). Furthermore, the study’s cross-sectional design makes it impossible to establish temporality, so it is unlikely that the observed associations are causal.

## 5. Conclusion

The study found that majority of the caregivers (84.2%) were active participants in the support groups for PLHIV in Tanzania. Participation was significantly associated with several factors, including the level of household hunger, place of residence-urban/rural, mental, or physical disability, age, marital status, and educational attainment. However, 15.8% of the caregivers were not participating in the PLHIV support groups, with the proportion rising to 29.7% among disabled caregivers.

To improve participation in the PLHIV support groups, tailored support should be provided to caregivers who are physically or mentally disabled and those who live in rural areas. Additionally, targeted support should be given to caregivers aged 30 or older, widowed, or unmarried, food insecure, and those with secondary education or more. These strategies could help increase participation rates among caregivers who may face specific barriers to accessing support groups. These findings highlight the need for targeted interventions to ensure that all caregivers LHIV are given the necessary support including linkage in PLHIV support groups to improve treatment outcomes.

## Data availability statement

The raw data supporting the conclusions of this article will be made available by the authors, without undue reservation.

## Ethics statement

The studies involving humans were approved by the Medical Research Coordinating Committee (MRCC) of the National Institute for Medical Research (NIMR) in Tanzania. The studies were conducted in accordance with the local legislation and institutional requirements. The participants provided their written informed consent to participate in this study.

## Author contributions

LK conceptualized the problem and critically reviewed the manuscript for intellectual content. AE participated in problem conceptualization, conducted statistical analysis, conducted literature review, wrote the first draft of the manuscript, and revised subsequent versions. JC prepared the data, participated in statistical analysis, and critically reviewed the manuscript for intellectual content. RM and TM conducted data management and critical review of the manuscript for intellectual content. AA participated in statistical analysis and critically reviewed the manuscript for intellectual content. ATe, AB, RF, ATa, GM, MB, EK, TV, and EL critically reviewed the manuscript for intellectual content. All authors contributed to the article and approved the submitted version.

## Funding

The ACHIEVE project is funded by the U.S. President’s Emergency Plan for AIDS Relief (PEPFAR) through the United States Agency for International Development (USAID). This work was conducted as a part of the authors’ responsibilities under their employment.

## Conflict of interest

The authors declare that the research was conducted in the absence of any commercial or financial relationships that could be construed as a potential conflict of interest.

## Publisher’s note

All claims expressed in this article are solely those of the authors and do not necessarily represent those of their affiliated organizations, or those of the publisher, the editors and the reviewers. Any product that may be evaluated in this article, or claim that may be made by its manufacturer, is not guaranteed or endorsed by the publisher.

## Figures and Tables

**Table:** 

ACHIEVE	Adolescents and Children HIV Incidence Reduction, Empowerment and Virus Elimination
AIDS	Acquired Immunodeficiency Syndrome
AGYW	Adolescent Girls and Young Women
ART	Antiretroviral Therapy
CCW	Community Case Worker
CIs	Confidence Intervals
CTC	Care and Treatment Centre
DREAMS	Determined, Resilient, Empowered, AIDS-free, Mentored and Safe
FCAA	Family and Child Asset Assessment
FSW	Female Sex Worker
HHS	Household Hunger Scale
HIV	Human Immunodeficiency Virus
ICC	Intra-class Correlation Coefficient
LHIV	Living with HIV
MRCC	Medical Research Coordinating Committee
NIMR	National Institute for Medical Research
NMNW	No Means No Worldwide
aOR	Adjusted Odds Ratio
OVC	Orphans and Vulnerable Children
PCA	Principal Component Analysis
PEPFAR	U.S. President’s Emergency Plan for AIDS Relief
PLHIV	People Living with HIV
SD	Standard Deviation
UHC	Universal Health Coverage
USAID	United States Agency for International Development
